# Sphingosine 1-Phosphate Receptor 5 (S1P5) Deficiency Promotes Proliferation and Immortalization of Mouse Embryonic Fibroblasts

**DOI:** 10.3390/cancers14071661

**Published:** 2022-03-25

**Authors:** Franck Talmont, Elodie Mitri, Christine Dozier, Arnaud Besson, Olivier Cuvillier, Anastassia Hatzoglou

**Affiliations:** 1 Institut de Pharmacologie et de Biologie Structurale, Université de Toulouse, CNRS, UPS, 31059 Toulouse, France; franck.talmont@ipbs.fr; 2 Molecular, Cellular and Developmental Biology (MCD), Centre de Biologie Intégrative, Université de Toulouse, CNRS, UPS, 31062 Toulouse, France; elodie.mitri@univ-tlse3.fr (E.M.); christine.dozier@univ-tlse3.fr (C.D.); arnaud.besson@univ-tlse3.fr (A.B.)

**Keywords:** sphingosine-1phosphate, S1P5, MEF immortalization, cell proliferation, cell spreading, cell migration, ERK, FAK

## Abstract

**Simple Summary:**

Sphingosine 1-phosphate (S1P) is a lipid metabolite involved in cell proliferation, survival or migration. S1P is a ligand for five high-affinity G protein-coupled receptors (S1P1-5), which differ in their tissue distribution, and the specific effects of S1P depend on the suite of S1P receptor subtypes expressed. To date, information regarding the role of S1P5 in cell proliferation is limited and ambiguous. Our results suggest that, unlike other S1P receptors, the S1P5 receptor has an anti-proliferative function. We found that S1P5 deficiency promotes cell immortalization and proliferation by controlling the spatial activation of ERK.

**Abstract:**

Sphingosine 1-phosphate (S1P), a bioactive lipid, interacts with five widely expressed G protein-coupled receptors (S1P1-5), regulating a variety of downstream signaling pathways with overlapping but also opposing functions. To date, data regarding the role of S1P5 in cell proliferation are ambiguous, and its role in controlling the growth of untransformed cells remains to be fully elucidated. In this study, we examined the effects of S1P5 deficiency on mouse embryonic fibroblasts (MEFs). Our results indicate that lack of S1P5 expression profoundly affects cell morphology and proliferation. First, S1P5 deficiency reduces cellular senescence and promotes MEF immortalization. Second, it decreases cell size and leads to cell elongation, which is accompanied by decreased cell spreading and migration. Third, it increases proliferation rate, a phenotype rescued by the reintroduction of exogenous S1P5. Mechanistically, S1P5 promotes the activation of FAK, controlling cell spreading and adhesion while the anti-proliferative function of the S1P/S1P5 signaling is associated with reduced nuclear accumulation of activated ERK. Our results suggest that S1P5 opposes the growth-promoting function of S1P1-3 through spatial control of ERK activation and provides new insights into the anti-proliferative function of S1P5.

## 1. Introduction

Sphingosine 1-phosphate (S1P) is a pleiotropic bioactive lipid found in organisms as diverse as yeast, flies, plants and mammals which has been implicated in a variety of cellular responses including cell growth and migration [1,2,3]. S1P exerts intracellular functions or acts extracellularly in an autocrine or paracrine manner through a family of five specific G protein-coupled receptors (S1PRs), called S1P1-5 [4]. Extracellular S1P signaling depends on which receptor subtypes are expressed [5], as S1P1-5 couple to different G proteins, and thus regulate various downstream signaling pathways with overlapping but also opposing functions [6].

Deregulation of the sphingolipid metabolism, such as altered S1P levels and/or the expression/activation of S1P receptors, has been linked to a variety of pathological conditions, including cancer, diabetes, fibrosis, inflammatory disorders, and multiple sclerosis [7,8]. Thus, S1P receptors contribute to tissue homeostasis and constitute challenging therapeutic targets. For example, FTY720 (Fingolimod), a sphingosine analog, which binds with similar affinities to S1P1 and S1P5, was approved by the US Food and Drug Administration (FDA) in 2010 for the treatment of relapsing-remitting multiple sclerosis [9]. It can be phosphorylated in vivo to form FTY720-phosphate (FTY720-P), an S1P mimetic interacting with S1P receptors but preferentially inducing internalization and degradation of S1P1 [10]. While the cellular functions of S1P1 and its internalization upon binding to S1P have been well studied, little is known about the roles of S1P5 and the underlying molecular mechanisms. Data on the cellular functions of S1P5 are indeed scarce and contradictory. A limited number of studies have examined the role of S1P5 in brain and natural killer cells, where S1P5 is highly expressed. S1P5 has been involved in oligodendrocyte survival and the maintenance of blood-brain barrier integrity, while in natural killer cells it controls egression to the lymphatic system [11,12,13]. S1P5 may also mediate S1P-induced autophagy in prostate cancer cells [14], while S1P5 overexpression is associated with decreased proliferation of esophageal cancer cells [15]. We recently reported that S1P regulates proper mitotic progression through the S1P5/AKT/PLK1 pathway [16,17].

In this study, we investigated the consequences of S1P5 loss in primary and immortalized mouse embryonic fibroblasts (MEFs). S1P5 deficiency reduces cellular senescence and promotes primary MEF immortalization. Our results suggest that S1P/S1P5 signaling decreases cell growth and opposes the growth-promoting functions of S1P/S1P1-3 through the spatial control of ERK activation, providing new insight into the anti-proliferative function of S1P5.

## 2. Materials and Methods

### 2.1. Cell Culture and Transfections

S1P5-deficient mice were described previously [11]. Primary MEFs isolated from wild-type (S1P5^+/+^) and S1P5^−/−^ embryos were a kind gift of Dr. Walzer (CIRI, Lyon, France). MEFs were maintained at 37 °C in high-glucose Dulbecco’s modified Eagles medium (DMEM; Gibco, LifeTechnologies, Carlsbad, CA, USA) supplemented with 10% fetal bovine serum (FBS) and penicillin/streptomycin (Sigma-Aldrich, St. Louis, MO, USA) as described previously [16]. Cells from different passages were used in different experiments, as indicated in figure legends. Immortalization of primary MEFs was performed according to the 3T3 protocol [18]. For wild-type MEFs, when proliferation ceased, cells were maintained without passage until regrowth became apparent (P16). Cultures from passage 18 onwards were considered immortalized. Immortalized MEFs were transfected using the Amaxa Nucleofector (Lonza, Basel, Switzerland).

CHO cells were obtained from the American Type Culture Collection and grown at 37 °C in a humidified atmosphere containing 5% CO_2_ in Dulbecco’s modified Eagles medium (4.5 g/L glucose, GlutaMAX, Gibco, LifeTechnologies, Carlsbad, CA, USA) supplemented with 10% heat-inactivated FBS and 100 U/mL penicillin, 100 μg/mL streptomycin. CHO cells were transfected with pcDNA-GFP-Hygro [19] or pcDNA-mS1P5-EGFP-N3 vectors, using Lipofectamine 2000 reagent (Invitrogen, Carlsbad, CA, USA) according to the manufacturer’s instructions. CHO cells stably expressing mS1P5-GFP or GFP were selected in presence of 500 μg/mL hygromycin B or G418, respectively, and drug resistant clones were isolated.

### 2.2. Plasmids, Reagents and Antibodies

The synthetic *Mus musculus* sphingosine 1-phosphate receptor 5, mS1P5 (Eurofins genomics, Ebersberg, Germany), was inserted into the pEGFP-N3 vector (Clontech) using XhoI and BamHI restriction enzymes. Reagents were obtained as follows: VPC23019 from Avanti Polar Lipids (Alabaster, AL, USA), JTE-013 from Tocris (R&D Systems, Minneapolis, MN, USA); Hygromycin B, G418 and Z-VAD-FMK from Invitrogen; S1P and FTY-720P were purchased from Enzo Life Sciences (Farmingdale, NY, USA). BrdU was obtained from Thermo Fisher Scientific (Waltham, MA, USA) and Phalloidin from Interchim (Montluçon, France). Rabbit polyclonal antibodies against p44/42 mitogen-activated protein kinase (ERK), phospho-p44/42 mitogen-activated protein kinase (phosphoERK), Akt, phospho-Akt (Ser473), FAK, phospho-FAK (Tyr575/577) were purchased from Cell Signalling Technology (Beverly, MA, USA). Mouse monoclonal antibodies against β-tubulin were from Sigma and against BrdU were from Thermo Fisher Scientific. Control (LT1017) and anti-S1P (LT1002) antibodies, a gift of Dr Sabbadini, were used as previously described [16,20].

### 2.3. [^0^S] GTPγS Binding Assay

To prepare membrane fractions, cells were harvested in phosphate buffer saline (PBS), frozen at least overnight at −80 °C, and then homogenized in ice-cold 50 mM Tris-HCl buffer, pH 7.5 using a Potter Elvehjem tissue grinder. The nuclear pellet was removed by centrifugation at 1000× *g* for 15 min at 4 °C. The total membrane fraction was collected after centrifugation of the supernatant at 100,000× *g* for 35 min at 4 °C. The membrane fraction was aliquoted and stored at −80 °C in 50 mM Tris-HCl, pH 7.4, and the protein concentration was determined by the Bradford method. The [^35^S] GTPγS binding assays were performed in polypropylene tubes in a buffer consisting of 20 mM HEPES pH 7.4, 100 mM NaCl, 5 mM MgCl_2_, 0.1% fatty acid-free BSA, and 10^−4^ M GDP. Membranes were incubated for 60 min at 30°C in the buffer supplemented with 5 µg saponin, 0.2 nM [^35^S] GTPγS, and 1 mM of the S1PRs agonist FTY720-P. The reaction was stopped by vacuum filtration through Whatman GF/B glass filters preincubated in buffer, which were then washed three times with 4 mL of ice-cold buffer without GDP. Membrane-bound radioactivity was determined by liquid scintillation counting (Packard, GMI Trusted laboratory Solutions, Ramsey, MN, USA) after overnight extraction of the filters in 4 mL of scintillation cocktail (Ecoscint A, National Diagnostics, Fisher Scientific).

### 2.4. Cell Proliferation and Survival Assays

The growth rate of immortalized MEF and CHO cells was monitored by seeding 15,000 cells and 30,000 cells per well, respectively, in triplicate in 12-well plates. At the indicated time points, the number of viable cells was counted by trypan blue staining. For cell survival studies, 20,000 MEFs were seeded in triplicate in 12-well plates and MTT assay (Sigma) was performed at the indicated time points.

### 2.5. BrdU Incorporation

Cells were seeded and after 24 h, and a cell culture medium containing 10 mM BrdU was added for 4 h. Cells were washed three times with PBS, fixed with 3.7% paraformaldehyde and permeabilized using 0.2% Triton X-100 PBS. DNA was denaturated using 2 M HCl for 30 min, and cells were immunostained using monoclonal anti-BrdU antibodies and rhodamine-conjugated secondary antibodies. BrdU-positive cells were counted to determine the proliferation rate and the results are presented as a percentage of BrdU-positive cells.

### 2.6. Colony Formation Assay

For low-density colony formation assays, cells were seeded in 6-well plates at 500 cells/well and maintained for 10 days under standard culture conditions. Cells were fixed and stained with crystal violet and photographed colonies were counted using the Image J software. Results are presented as the number of colonies per well.

### 2.7. Cell Senescence Assay

Senescence-associated-β-galactosidase (SA-β-Gal) activity identifies senescent cells in culture [21]. SA-β-Gal activity was measured with the senescence β-Gal kit (Cell Signalling Technology, Beverly, MA, USA) following the manufacturer’s instructions. Briefly, MEFs from different passages were fixed and SA-β-Gal activity was detected at pH 6.0. Cells with a distinctive blue color were counted and the percentage of senescent cells was determined (number SA-β-Gal positive cells/total number of cells).

### 2.8. Cell Spreading Assay

MEFs were seeded on glass coverslips and allowed to spread for 0.5, 1, 2 or 3 h at 37 °C in the presence of complete media. Cells were fixed by adding PFA directly to the culture medium at a final concentration of 2% for 20 min at 37 °C and stained with Phalloidin-Cy3 (1/400) for 45 min to visualize cellular outlines and with Hoechst 33,342 to label DNA. Images were captured on a Nikon 90i Eclipse microscope (Nikon Instruments, Melville, NY, USA) using a 20× objective for quantifications or 40× objective for representative images. Cell surface boundaries were outlined for *n* = 100 randomly chosen cells and cell surface measurements were performed using the ImageJ software.

### 2.9. Cell Adhesion Assay

For cellular adhesion experiments, 30,000 cells were seeded in 24-well plates uncoated or coated with fibronectin (Sigma; 10 µg/mL fibronectin for 1 h at 37 °C) and incubated at 37 °C for the indicated times. Cells were washed three times with PBS to remove nonadherent cells, fixed with 3.5% formaldehyde, and stained with crystal violet after two washes. The cells were lysed and absorbance was measured at 595 nm. S1P5^+/+^ cell adhesion at 30 min was normalized to 1, and results are presented as adhesion fold change.

### 2.10. Transwell Migration Assay

Equal numbers of S1P5^−/−^ and S1P5^+/+^ MEFs were seeded in 8-µm pore transwell cell culture inserts (BD Biosciences, France) and complete culture medium was added to the lower compartment. Cells were allowed to migrate for 8 h at 37 °C. Cells on the top side of the membrane were removed while cells that migrated to the bottom of the well were fixed and stained with crystal violet. The filters were then imaged with a Leica inverted microscope. Representative images were randomly captured for each insert and used to manually count the number of cells present. The results were expressed as relative migration normalized to wild-type cells.

### 2.11. Immunocytochemistry

CHO cells stably expressing mS1P5-GFP or GFP alone were fixed with 4% paraformaldehyde for 10 min at room temperature and stained for DNA with DAPI prior to observation. Images were acquired on an IX73 Olympus microscope equipped with a 60X oil immersion objective (numerical aperture 1.4).

Morphological characterization of wild-type and S1P5^−/−^ immortalized MEFs was performed as previously described [22]. Briefly, cells were grown on coverslips for 24 h and stained with crystal violet. The maximal length (L) and width (W) of cells were measured using the Image J software, and cell elongation was calculated as L/W.

For subcellular localization of activated ERK, MEFs were grown on coverslips in culture medium as indicated in figure legends. Cells were washed once with PBS, fixed with 2% PFA for 20 min at 37 °C, and permeabilized with 0.1% Triton X-100 PBS for 5 min. Cells were incubated with rabbit antibodies against phospho-ERK1/2 at 4 °C overnight. Coverslips were washed three times with PBS and incubated with Alexa-488 anti-rabbit secondary antibodies and Phalloidin-Cy3 to stain F-actin at room temperature for 45 min. DNA was stained with Hoechst 33342. For quantification of phospho-ERK1/2 per nucleus, images were acquired on a Nikon 90i Eclipse microscope using a 20× objective at the same exposure settings. Results are presented as fluorescence intensity per square micrometer.

### 2.12. Immunoblotting

Cells were lysed in an LDS sample buffer (NuPAGE, Novex, Life Technologies, Carlsbad, CA, USA), sonicated, and boiled at 96 °C for 3 min. Proteins were separated on 4–20% gradient SDS-PAGE (Bio-Rad, Hercules, CA, USA) and transferred to polyvinylidene difluoride membrane (Immobilon-P, Millipore, Burlington, MA, USA). Membranes were blocked in TBS-T (Tris Buffered Saline with 0.1% Tween 20) containing 5% Bovine Serum Albumin (Euromedex, Souffelweyersheim, France) or 5% skim milk and incubated with primary antibodies overnight at 4 °C. Membranes were washed and then incubated at room temperature with Horseradish peroxidase-conjugated secondary antibodies (Jackson ImmunoResearch, Ely, UK). After washing, detection was performed by chemiluminescence (Clarity^TM^ Western ECL substrate, Bio-Rad). Image acquisition and quantification of immunoblots were performed with a Fusion Solo X chemiluminescence imaging system using the Evolution Capture software (Vilber Lourmat, Marne La Vallée, France).

### 2.13. Data and Statistical Analyses

Data are presented as means ± SEM of at least three independent experiments. Statistical analyses were performed using GraphPad Prism (version 4.0c for Macintosh, GraphPad Sofware Inc., San Diego, CA, USA). Statistical significance for independent groups was assessed using a Student’s *t*-test. *p* < 0.05 was considered a statistically significant difference.

## 3. Results

### 3.1. S1P5 Deficiency Confers Resistance to Cellular Senescence and Promotes MEFs Immortalization

**Figure 1:**
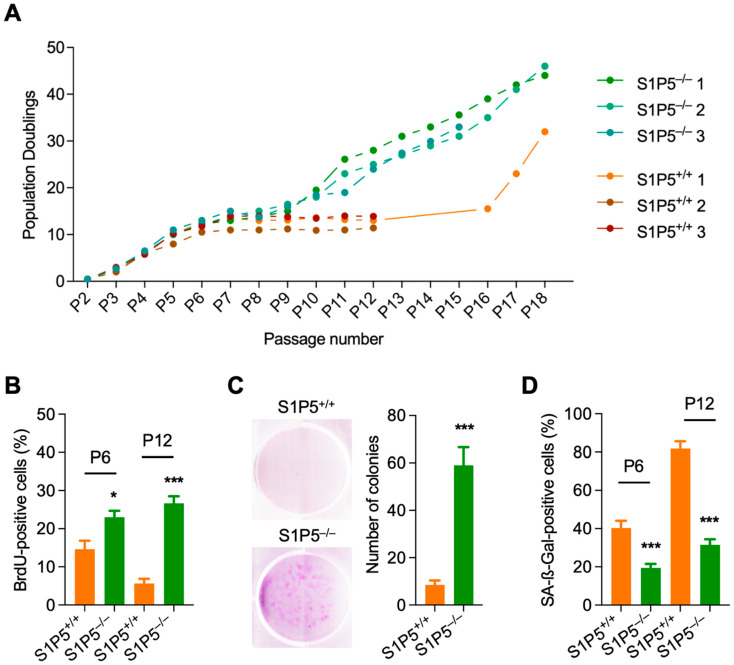
Primary S1P5^−/−^ MEFs are resistant to cellular senescence and prone to immortalization. (**A**), population doublings of S1P5^+/+^ and S1P5^−/−^ primary MEFs over serial passaging according to 3T3 protocol. S1P5^+/+^ and S1P5^−/−^ MEFs were each isolated from three different embryos. (**B**), wild-type and S1P5-deficient MEFs at passage six (P6) and passage 12 (P12), were labeled with BrdU for 3 h. The incorporated BrdU was visualized by immunofluorescence. DAPI staining was used to visualize nuclei. Data represent means ± SEM of three different clones performed in triplicates (*, *p* < 0.0114, ***, *p* < 0.0007). (**C**), colony formation capacity of S1P5^+/+^ and S1P5^−/−^ MEFs at passage 6 (P6). Wild-type and S1P5-deficient MEFs were plated in a 6 well-plate and 10 days later cells were fixed and stained with crystal violet to visualize colony formation. Representative images are shown in the left panel. Right panel: means of colony number ± SEM of three different clones performed in triplicates (***, *p* < 0.0001). (**D**), S1P5^+/+^ and S1P5^−/−^ MEFs at passage 6 (P6) and passage 12 (P12), were stained for SA-β-Gal activity. SA-β-Gal-positive cells were counted in more than five fields, and results represent the means ± SEM of three different clones performed in triplicates (***, *p* < 0.0001).

To investigate the effect of S1P5 loss on MEF proliferation, freshly isolated MEFs from S1P5 wild-type (S1P5^+/+^) and S1P5 deficient (S1P5^−/−^) mice were cultured using the 3T3 protocol [23]. Thirty thousand cells were seeded in 10 cm dishes and re-plated twice a week to prevent MEFs from reaching contact inhibition. As expected, primary MEFs exit the cell cycle as the number of passages increases, resulting in a growth arrest phase on average at passage six to seven (Figure 1A) [23]. However, S1P5^−/−^ MEFs resumed proliferating after a short growth stasis (passage seven to nine), indicating the emergence of immortalized clones [24] (clone S1P5^−/−^ 3 was lost at passage 15 after contamination). The growth of wild-type MEFs decreased over time and only one out of the three starting lines eventually became immortalized at passage 16 (Figure 1A). Consistent with these observations, S1P5^−/−^ MEFs exhibited higher BrdU incorporation at passages six and 12 compared to wild-type S1P5^+/+^ MEFs (Figure 1B). In addition, clonogenicity assays showed that S1P5-deficient cells at low passage formed more colonies than wild-type cells (Figure 1C). These results suggest that S1P5 deficiency might protect cells from cellular senescence. Indeed, loss of S1P5 significantly reduced the percentage of SA-β-gal-positive cells compared to wild-type MEFs; however, despite their ability to grow, some S1P5^−/−^ cells retained some features of cell senescence such as β-gal staining (Figure 1D) [25]. Taken together, these results indicate that S1P5-deficiency promotes MEF immortalization and protects them from cellular senescence.

### 3.2. S1P5 Loss Results in Cell Elongation and Impaired Adhesion, Spreading and Migration

**Figure 2:**
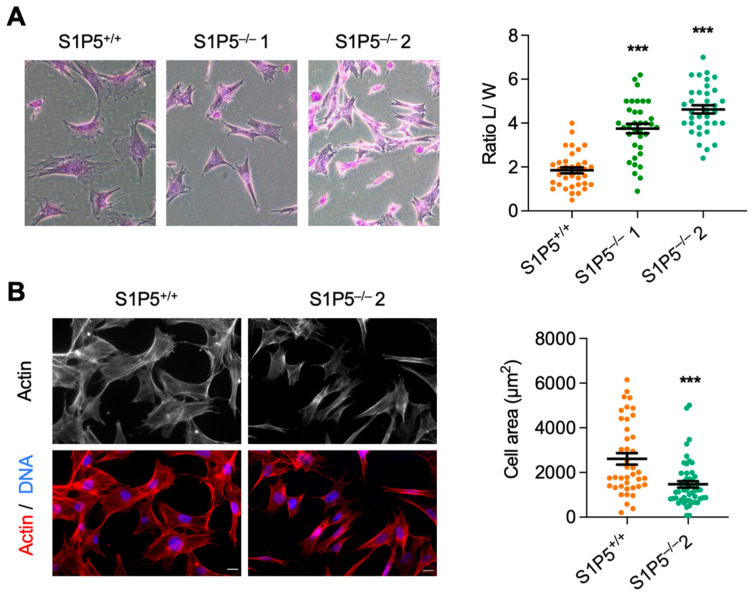
Morphological characterization of S1P5^+/+^ and S1P5^−/−^ immortalized clones. (**A**), phenotype of immortalized MEFs. Representative phase contrast images from low confluence cultures of S1P5^+/+^ and S1P5 ^−/−^ cells at passage 18 after fixation and staining with crystal violet are shown in the left panel. Right panel: quantification of the ratio of maximal length (L) versus cell width (W) from *n* = 30 cells per condition from four independent experiments performed between passages 18 to 22. Each dot represents a cell (***, *p* < 0.0001). (**B**), cell area and actin organization in S1P5^−/−^ cells. S1P5^+/+^ and S1P5^−/−^ 2 MEFs were plated on coverslips and 24 h later were fixed and stained for F-Actin (Phalloidin-red) and DNA (Hoechst 33,342-blue). The left panel shows representative images. Scale bar, 10 μm. The right panel shows the quantification of the cell area in µm^2^ based on F-Actin staining. The mean surface area was calculated using ImageJ software. The data are represented in a dot plot, where each point represents one cell and the black lines indicate means ± SEM (***, *p* < 0.0001; S1P5^+/+^, 2612 ± 255, *n* = 41; S1P5^−/−^ 1467 ± 147, *n* = 50).

S1P5 deficiency was associated with changes in cell morphology. S1P5^−/−^ cells were more refractile and exhibited a decrease in size compared to wild-type MEFs (Figure 2A, left panel). Loss of S1P5 expression resulted in cell elongation, which was quantified by measuring the ratio of their maximal length to width, compared with wild-type MEFs (Figure 2A, right panel).

**Figure 3:**
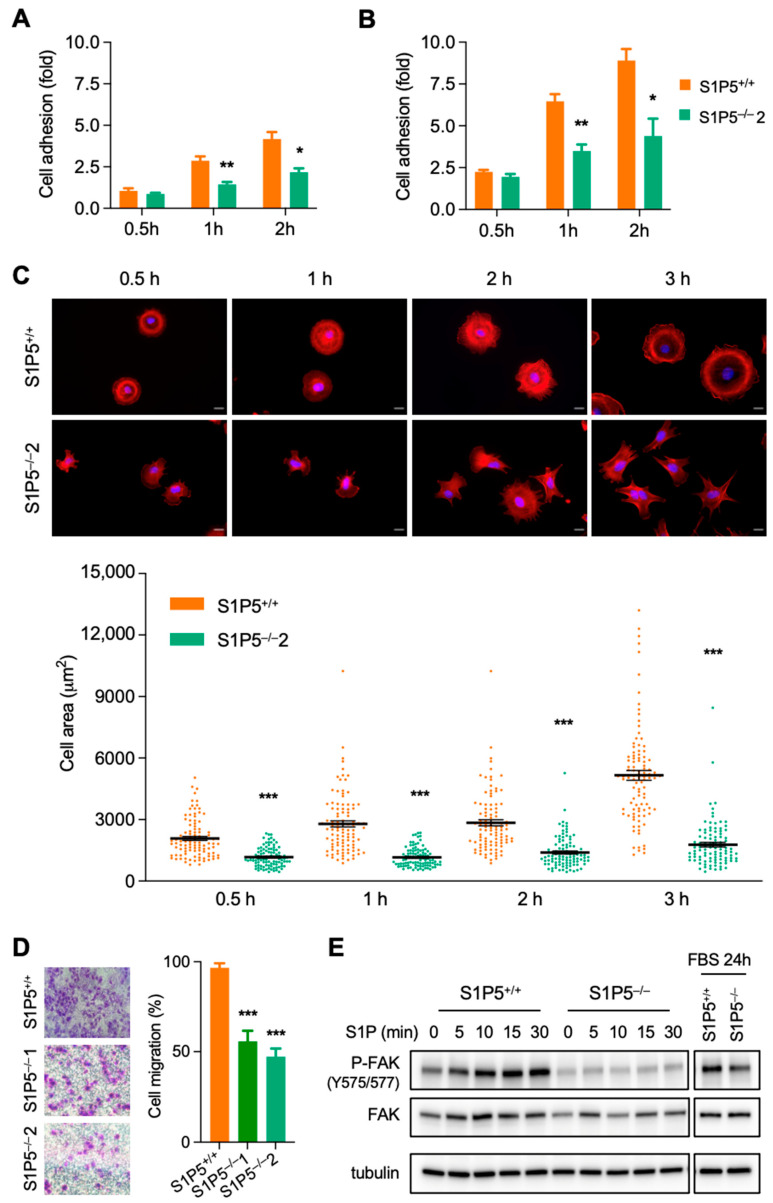
Impaired adhesion, spreading and migration in S1P5^−/−^ cells. S1P5^+/+^ and S1P5^−/−^ MEFs were plated in non-coated (**A**) or fibronectin-coated (**B**) 24-well plates and incubated for 0.5, 1, and 2 h. Cells were washed and the adherent cells were stained with crystal violet, and the staining intensity was quantified by spectrophotometry at 595 nm. The results represent fold cell adhesion (of wild-type cells) and are means ± SEM of three independent experiments (**, *p* < 0.0083; *, *p* < 0.0149; **, *p* < 0.0073; *, *p* < 0.0226). (**C**), cell spreading and actin organization in S1P5^−/−^ cells. S1P5^+/+^ and S1P5^−/−^ 2 MEFs were plated on coverslips for the indicated times, fixed and stained for F-Actin (Phalloidin-red) and DNA (Hoechst 33,342-blue). The upper panel shows representative images. Scale bar: 10 μm. The lower panel shows the quantification of the cell area (in µm^2^) based on F-Actin staining. The mean surface area from 100 individual cells was calculated using the ImageJ software. The data are shown as dot plot, where each point represents one cell and the black lines indicate means ± SEM (***, *p* < 0.0001). (**D**), equal numbers of S1P5^+/+^ and S1P5^−/−^ MEF cells were seeded in 8-µm pore Transwell cell culture inserts and incubated for 8 h. The cells that migrated to the bottom of the well were fixed and stained with crystal violet. The left panel shows representative images and the right panel the relative migration (expressed as percentage of control cells). The means ± SEM from four experiments is shown (***, *p* < 0.0001). (**E**), impaired FAK phosphorylation in S1P5^−/−^ MEFs. S1P5^+/+^ and S1P5^−/−^ cells were serum-starved for 4 h and then treated with S1P (1 µM) for the indicated times or grown in medium containing 10% FBS for 24 h (right panel). Total cell extracts were analyzed by SDS/PAGE with antibodies against phosphorylated FAK, FAK and tubulin.

Furthermore, cell surface area was significantly reduced in S1P5^−/−^ cells compared to wild-type MEFs (Figure 2B). In association with these morphological changes, phalloidin staining revealed changes in F-actin distribution, with pronounced cortical actin in S1P5^−/−^ cells (Figure S1A). In addition, vinculin staining showed that there were fewer focal adhesion sites in S1P5^−/−^ MEFs in agreement with their reduced size (Figure S1B). Next, we compared the adhesion properties of S1P5^+/+^ and S1P5^−/−^ cells on glass coverslips coated or not with fibronectin at 0.5, 1 and 2 h after plating. At 1 and 2 h after plating, the number of S1P5^−/−^ cells adhering to uncoated or fibronectin-coated coverslips was significantly reduced compared to wild-type MEFs (Figure 3A,B, respectively). After adhering to the extracellular matrix (ECM), the cells spread out and acquired a flattened morphology [26]. As expected, wild-type MEFs spread isotropically, whereas S1P5^−/−^ cells spread anisotropically, with membrane protrusions appearing within 30 min (Figure 3C, upper panel). Cell boundaries were visualized by phalloidin staining and the cell spreading was obtained by measuring the cell surface area. At all times studied, S1P5^−/−^ cells spread significantly less than wild-type MEFs, and at 3 h, the cell area of S1P5^−/−^ cells was reduced by 75% of that of S1P5^+/+^ MEFs (Figure 3C; 5157 ± 237 µm^2^ versus 1777 ± 110 µm^2^). Because cell spreading can affect cell migration, MEFs motility was assessed by transwell assays. As shown in Figure 3D, the loss of S1P5 expression significantly inhibited MEF cell migration.

Focal adhesion kinase (FAK) plays a crucial role in transducing signals initiated by the interaction between integrins and ECM proteins, which in turn regulate cell adhesion, spreading and migration [27]. The impaired spreading and migration of S1P5^−/−^ MEFs suggests a potential role of S1P5 in FAK activation. When serum-starved wild-type cells were stimulated with S1P, FAK was rapidly phosphorylated and activation lasted for at least 30 min (Figure 3E). In contrast, S1P5^−/−^ cells did not activate FAK upon S1P treatment, suggesting that S1P induces FAK activation via S1P5 (Figure 3E). Interestingly, under basal culture conditions (24 h in 10% FBS-containing medium), FAK phosphorylation was reduced in S1P5^−/−^ MEFs compared to wild-type S1P5^+/+^ cells (Figure 3E and Figure S2), confirming that S1P5 participates in inducing FAK phosphorylation induced by mitogenic factors in FBS. Taken together, these results indicate that the S1P/S1P5 signaling pathway promotes FAK activation and promotes cell spreading and motility.

### 3.3. Loss of S1P5 Promotes Cell Proliferation

**Figure 4:**
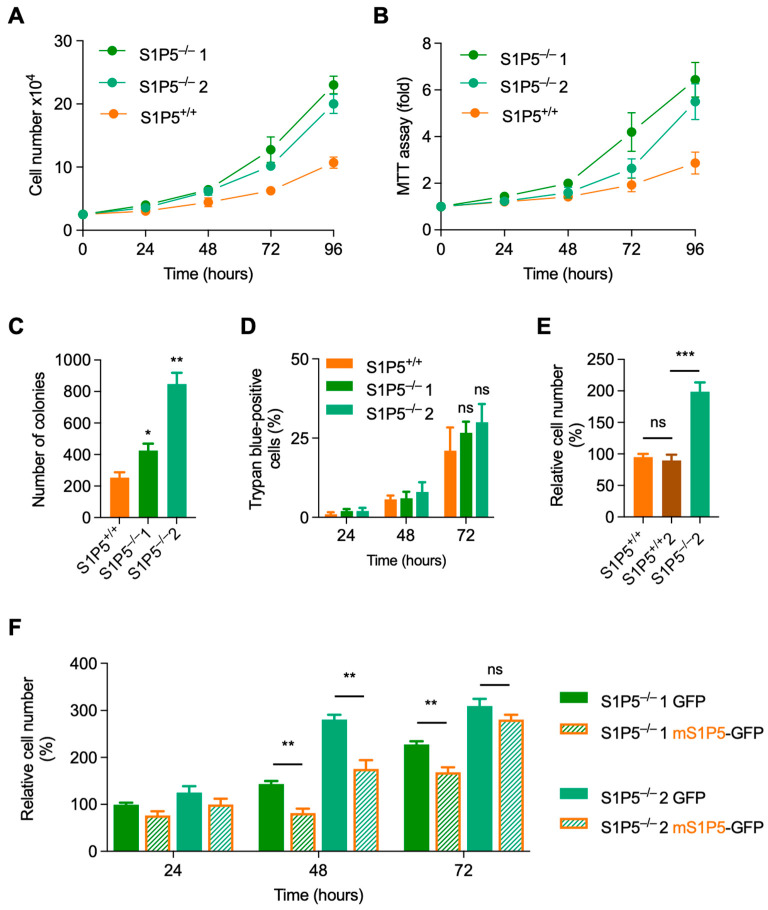
Growth properties of immortalized S1P5-deficient cells. (**A**), S1P5^+/+^ and S1P5 ^−/−^ cells were plated at equal cell number and the cell number was counted daily over four days. Results are representative from four independent experiments performed in triplicates from MEFs at passages 18 to 22. (**B**), cell survival of S1P5^+/+^ and S1P5^−/−^ MEFs (passages 18–22) was assessed by MTT assays at the indicated times after seeding. Results are representative from three independent experiments performed in triplicates. (**C**), colony formation capacity of immortalized S1P5^+/+^ and S1P5^−/−^ MEFs between passages 18–22. Low number of cells were plated and allowed to grow for 10 days. Cells were then fixed and stained with crystal violet to visualize colony formation. Graph shows the means of colony number ± SEM from three independent experiments performed in triplicates (*, *p* < 0.0357; **, *p* < 0.0017). (**D**), cell death/viability was evaluated by trypan blue staining. S1P5^+/+^ and S1P5^−/−^ cells were plated at equal cell number and the number of trypan blue-positive cells was counted at the indicated time points. Results are means ± SEM from three independent experiments performed in triplicates. (ns, not significant). (**E**), equal number of S1P5^+/+^, S1P5^+/+^ clone 2 (S1P5^+/+^ 2) and S1P5^−/−^ 2 cells were plated and the cell number was counted 72 h later. Results represent means of the relative number of cells ± SEM from three independent experiments performed in triplicates (***, *p* < 0.0007; ns, *p* = 0.6279). (**F**), S1P5 rescues the S1P5^−/−^ phenotype. Immortalized MEFs S1P5^−/−^ clone 1 and clone 2 were transiently transfected with empty vector (GFP) or a vector containing mS1P5-GFP. The total cell number was evaluated at different post transfection times. Results represent means of the relative number of cells ± SEM of three independent experiments performed in triplicates (**, *p* < 0.0058; **, *p* < 0.0099; **, *p* < 0.0079; ns, *p* = 0.1879).

The proliferation of immortalized S1P5^+/+^ and S1P5^−/−^ MEFs (passages 18–22) was assessed by counting by trypan blue negative cells. As shown in Figure 4A, S1P5^−/−^ MEFs showed a growth advantage compared to wild-type cells over a four-day period. Cell cycle length was approximated to the time of cell population doubling. In agreement with the literature, the average cell cycle length for wild-type MEFs was 24 h, whereas it was approximately 18 h in S1P5^−/−^ MEFs. Furthermore, S1P5^−/−^ cells had a survival advantage over wild-type cells, as shown by MTT (Figure 4B) and colony formation assays (Figure 4C), although there was no significant difference in cell death monitored by trypan blue staining (Figure 4D). During the course of this work, a second wild-type S1P5^+/+^ immortalized line (S1P5^+/+^ 2) was generated in the laboratory and had similar proliferation characteristics as the first one. Both wild-type clones proliferated slower than S1P5^−/−^ cells (Figure 4E). Overall, these results suggest that immortalized S1P5^−/−^ MEFs proliferate more rapidly than S1P5^+/+^ cells. To confirm the role of S1P5 in regulating cell proliferation, S1P5 was reintroduced in S1P5^−/−^ MEFs. Because we were unable to obtain MEFs stably expressing S1P5, growth properties were assessed in cells transiently expressing mouse S1P5 fused to GFP (mS1P5-GFP). Expression of mS1P5-GFP in S1P5^−/−^ cells at least partially reduced proliferation compared to control GFP transfected cells (Figure 4F). However, this partial effect could be due to the low transfection rate (30–40%) and the fact that untransfected cells continued to proliferate at an increased rate and rapidly dominated the cultures. Since both S1P5^−/−^ clones show similar growth properties, clone 2, designated S1P5^−/−^, was used for further studies. In conclusion, these results strongly suggest that the S1P5 receptor inhibits cell proliferation.

**Figure 5:**
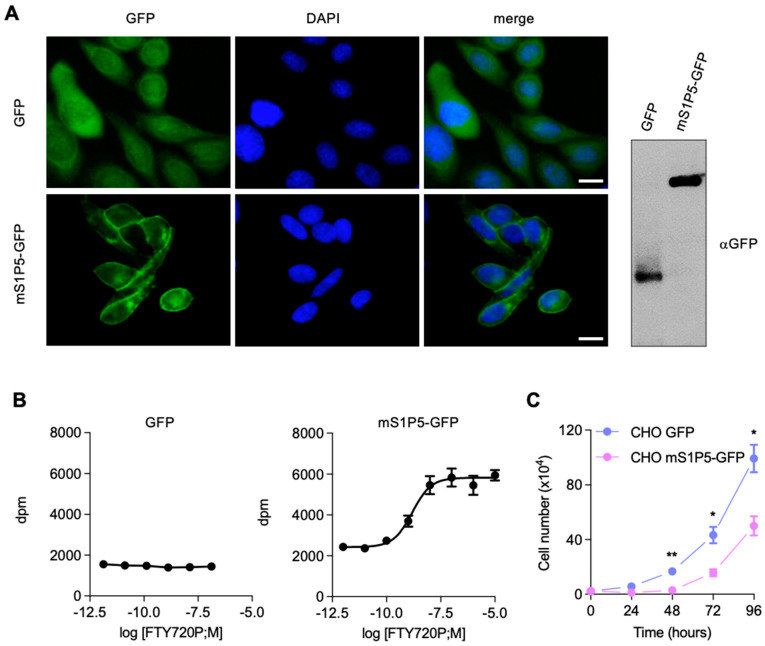
S1P5 induces growth arrest. (**A**), representative images showing CHO cells stably expressing GFP or mS1P5-GFP and stained for DNA with DAPI. Scale bar: 10 μm. Right panel shows GFP and mS1P5-GFP levels in CHO cells analyzed by Western blot using antibodies against GFP. (**B**), pharmacological characterization of mS1P5R-GFP. Agonist stimulation of CHO cells ectopically expressing mS1P5-GFP or GFP alone. Membrane fractions were isolated and stimulated with increased concentrations of S1P or FTY720-P. The graph is representative of two independent experiments performed in duplicate. (**C**), growth properties of CHO cells stably expressing mS1P5R-GFP or GFP. CHO mS1P5-GFP or CHO GFP cells were plated at equal cell number and the cell number was counted daily over a period of four days. Growth curves show means ± SEM from three independent experiments performed in triplicates (**, *p* < 0.0021; *, *p* < 0.0134; *, *p* < 0.0157).

We have previously shown that MEFs express all S1P receptors [16], which makes it difficult to study the specific functions of S1P5. In contrast, CHO cells do not express any S1P receptors and thus are a suitable model to dissect the cellular effects of single S1P receptors [28,29]. To define how S1P5 controls proliferation, CHO cells stably expressing either mS1P5-GFP or GFP as a control were generated (Figure 5A). S1P5-GFP exhibited a punctate localization in the cytoplasm and plasma membrane, whereas GFP was diffusely present in the nucleus and cytoplasm, as expected (Figure 5A). To verify that GFP-coupled S1P5 was functional, the ability of mS1P5-GFP to bind to a specific agonist (FTY720-P) was measured (EC50 = 3.01 ± 1.12 nM), whereas no binding was observed in control GFP cells (Figure 5B). In cell proliferation assays, cells expressing mS1P5-GFP showed considerably reduced cell numbers over four days compared with GFP control cells, confirming that S1P5 has an anti-proliferative activity when overexpressed in CHO cells (Figure 5C).

### 3.4. S1P5 Decreases Cell Proliferation in a Ligand Dependent Manner

**Figure 6:**
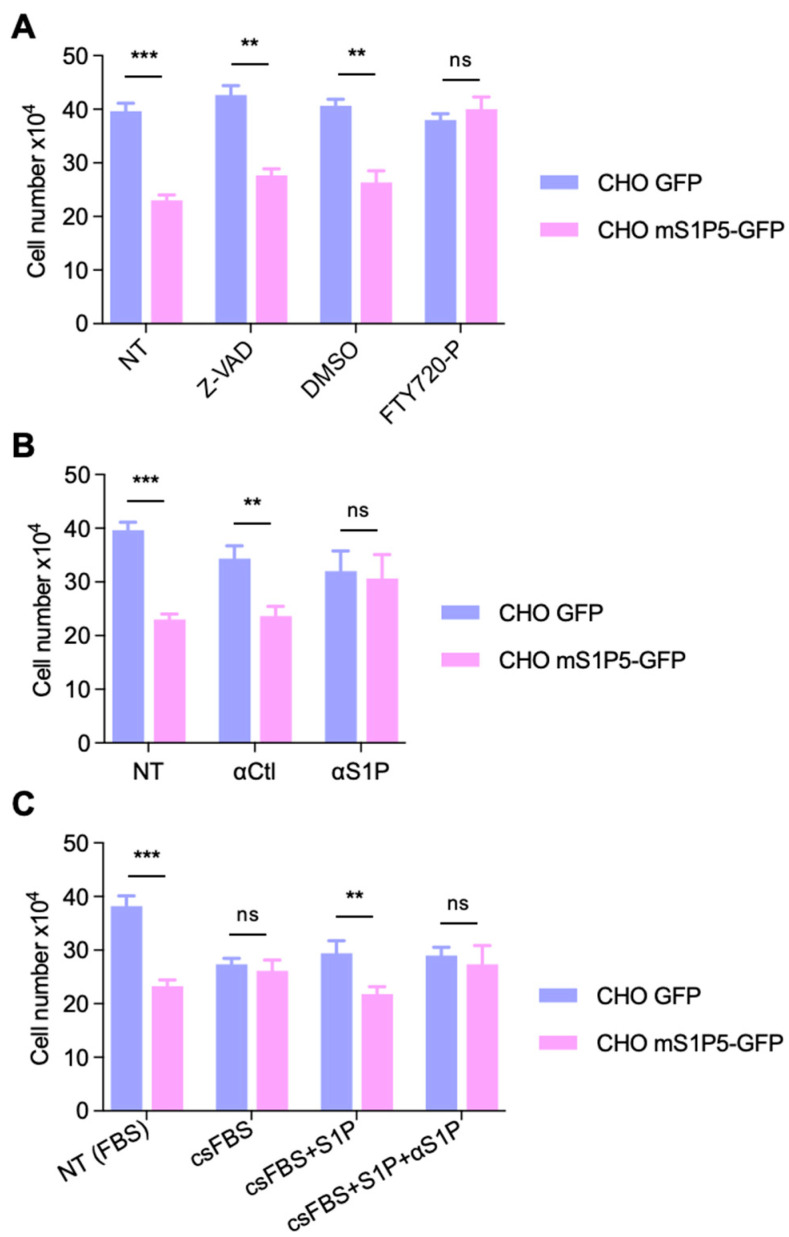
S1P5-induced growth inhibition is ligand-dependent. (**A**), CHO cells expressing mS1P5-GFP or GFP alone were plated at 20,000 cells/well in 12 well-plates. After 16 h, cells were left untreated (NT) or treated with Z-VAD (10 µM), vehicle (DMSO), or FTY720-P (1 µM), and the cell number was counted 48 h post treatment. Graphs show means ± SEM from three independent experiments performed in triplicate (***, *p* <0.0007; **, *p* < 0.0022; **, *p* < 0.0045; ns, *p* = 0.6309). (**B**), mS1P5-GFP or GFP expressing CHO cells were plated as in (A) and treated with control antibodies (α-Ctl) or antibodies against S1P (α-S1P). Cells were counted 48 h post treatment. Graphs show means ± SEM from four independent experiments performed in triplicates (*** *p* < 0.0007; ** *p* < 0.0048; ns, *p* = 0.8298). (**C**), CHO cells expressing mS1P5-GFP or GFP alone were plated as in (**A**). After 16 h, cells were washed and treated in DMEM supplemented with delipidated FBS (csFBS) for 24 h in the absence or in the presence of S1P (1 µM) or S1P and α-S1P. Cells grown in medium containing 10% FBS were also counted (FBS). Results show cell number 48 h post treatment and are means ± SEM from at least three independent experiments performed in triplicate (***, *p* < 0.0001; ns, *p* = 0.6309; **, *p* < 0.0033; ns, *p* = 0.6870).

We next investigated the molecular mechanism underlying S1P5-mediated cell growth inhibition. Indeed, S1P1-5 are activated by intracellular and extracellular S1P, but may also have ligand-independent roles. The decrease in cell number in mS1P5-GFP compared to GFP cells was not due to apoptosis because experiments performed in the presence of the pan-caspase inhibitor Z-VAD gave similar results (Figure 6A).

FTY720-P is a potent S1P receptor ligand that induces internalization, sequestration or degradation of S1P receptors, thus acting as an inhibitor when present for long periods of time [10]. S1P5-induced inhibition of proliferation was rescued when cells were grown in the presence of FTY720-P (Figure 6A). Because S1P is present in the serum [30], we used Sphingomab, a high-affinity anti-S1P (αS1P) monoclonal antibody that neutralizes extracellular S1P [16,31]. The addition of αS1P to the culture medium specifically reversed the S1P5-induced growth phenotype compared to control antibodies, indicating that extracellular S1P inhibits CHO cell growth via activation of S1P5 (Figure 6B). Furthermore, control (GFP) and S1P5 (mS1P5-GFP) expressing cells grown in media containing delipidated charcoal-stripped FBS (csFBS) had similar growth properties, confirming that S1P present in the serum is required for S1P5-induced growth inhibition. The addition of exogenous S1P restored the inhibition of proliferation of S1P5-expressing cells cultured in csFBS, and this effect was prevented by neutralizing αS1P (Figure 6C). Taken together, these results indicate that S1P/S1P5 signaling results in growth inhibition.

### 3.5. The S1P/S1P5 Axis Negatively Regulates MEF Proliferation

**Figure 7:**
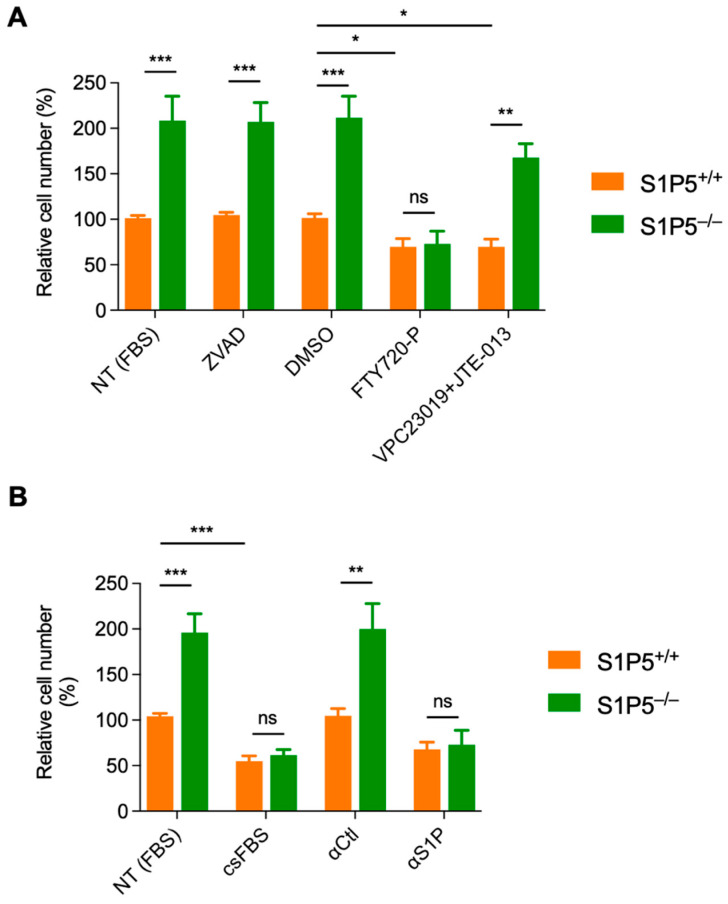
Knockdown of S1P5 promotes cells growth in a S1P dependent manner. (**A**), S1P5^+/+^ and S1P5^−/−^ cells were plated at 20,000 cells/well and 16 h later, cells were treated with vehicle (DMSO), Z-VAD (10 µM), FTY720-P (1 µM) or VPC23019 (10 µM) + JTE-013 (10 µM). Results show relative cell number expressed as a percentage of control non-treated cells ± SEM from more than three independent experiments performed in triplicates (***, *p* < 0.0004; ***, *p* < 0.0007; ***, *p* < 0.0006; ns, *p* = 0.8656; **, *p* < 0.0049; *, *p* < 0.0240; *, *p* < 0.0217). (**B**), S1P5^+/+^ and S1P5^−/−^ cells cultured as in (A) and were either grown in DMEM containing delipidated FBS (csFBS) or in complete medium containing FBS in the presence of control antibodies (α-Ctl) or antibodies against S1P (α-S1P). Cells were counted 48 h post treatment. Results show the relative cell number expressed as a percentage of control non-treated cells ± SEM from three independent experiments performed in triplicate (***, *p* < 0.0001; ns, *p* = 0.4685; **, *p* < 0.0031; ns, *p* = 0.7922; ***, *p* < 0.0005).

Because all S1PRs are expressed in MEFs [16], we studied the involvement of the S1P/S1P5 axis in MEF proliferation in the context of the presence of all the other S1PRs. As in CHO cells, the cell growth advantage induced by loss of S1P5 in MEFs was independent of apoptosis since Z-VAD had no effect (Figure 7A). When cells were grown in the presence of the S1P1-5 functional antagonist FTY720-P, wild-type and S1P5^−/−^ cells showed similar proliferation rates. Importantly, FTY720-P significantly reduced the number of S1P5^+/+^ cells compared to untreated cells (DMSO), suggesting that S1P1-4 receptors positively regulate MEF proliferation (Figure 7A).

To examine the involvement of S1P1-3 receptors in MEF proliferation, these assays were performed in presence of the pharmacological inhibitors VPC23019 (specific for S1P1 and S1P3) and JTE-013 (specific for S1P2). Inhibition of S1P1-3 significantly decreased cell growth in wild-type cells compared to untreated cells, (69% vs. 100%; Figure 7A) suggesting that one or more S1P1-3 receptors promote cell proliferation in immortalized MEFs. In contrast, in the presence of VPC23019 and JTE-013, S1P5^−/−^ cells continued to proliferate significantly faster than S1P5^+/+^ cells, further confirming that S1P5 has an anti-proliferative effect.

We next investigated whether extracellular S1P controlled MEF cell growth via S1P5. When MEFs were grown in the absence of extracellular S1P (delipidated csFBS) or in the presence of extracellular S1P neutralizing antibodies (αS1P), S1P5^+/+^ and S1P5^−/−^, cells had similar proliferation rates, and the growth advantage of S1P5^−/−^ cells was reversed (Figure 7B). Overall, these results strongly suggest that S1P inhibits MEF proliferation via S1P5.

### 3.6. Loss of S1P5 Leads to Persistent Activation of ERK and Its Nuclear Localization

**Figure 8:**
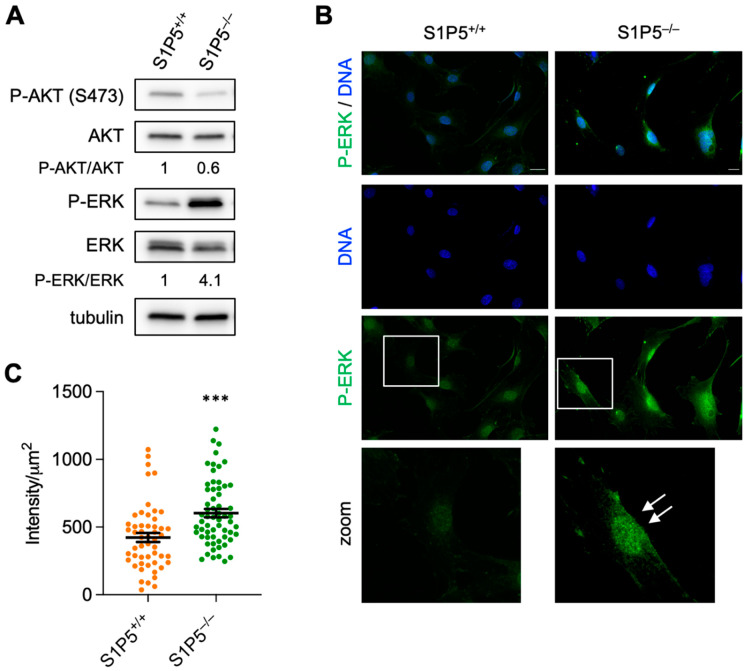
S1P5 reduces nuclear P-ERK localization. (**A**), S1P5^+/+^ and S1P5^−/−^ cells were grown in medium containing 10% FBS for 24 h. Total cell extracts were analyzed by SDS/PAGE with antibodies against the indicated proteins. (**B**), S1P5^+/+^ and S1P5^−/−^ MEFs were cultured as in (**A**), fixed and stained for phospho-ERK (a-ERK-green), DNA (Hoechst 33,342-blue) and actin (phalloidin-red). Representative images are shown in the upper panel. Scale bar: 10 μm. (**C**), quantification of nuclear phospho-ERK staining based on anti-ERK and Hoechst 33,342 staining. Images were taken with a 20× objective and the same exposure settings were used for all fluorescence quantifications. Results are expressed as fluorescence intensity per µm^2^ (***, *p* < 0.0001; S1P5^+/+^, 423.3 ± 33.30, *n* = 52; S1P5^−/−^ 603.6 ± 30.77, *n* = 61).

Since MAPK and Akt signaling are crucial for MEF proliferation, we studied the phosphorylation levels of ERK and Akt under basal culture conditions. Akt phosphorylation was decreased in S1P5^−/−^ cells grown for 24 h in medium supplemented with 10% FBS, whereas under the same culture conditions, ERK phosphorylation was increased in S1P5^−/−^ cells compared to S1P5^+/+^ MEFs, suggesting that S1P5 negatively regulates ERK activation by mitogenic factors present in FBS (Figure 8A). Phosphorylated ERK shuttles between the cytoplasm and the nucleus, and nuclear phospho-ERK has been shown to promote MEF proliferation, whereas cytoplasmic phospho-ERK induces cell cycle arrest and growth inhibition [32]. As temporal but also spatial control of ERK activation is crucial for cell fate decisions, we investigated whether the increased ERK phosphorylation in S1P5-deficient cells correlates with its nuclear localization. As seen in Figure 8B, phosphorylated ERK accumulated in the nuclei of S1P5^−/−^ cells compared to proliferating S1P5^+/+^ MEFs. Nuclear boundaries were defined by Hoechst 33,342 staining and the phospho-ERK staining intensity per nuclei was quantified. The intensity of nuclear phosphorylated ERK was significantly elevated in S1P5^−/−^ cells compared to wild-type cells (Figure 8C; 604 ± 31 versus 423 ± 33 respectively). These results suggest that S1P5 downregulates FBS-induced activation of ERK by controlling the spatial ERK phosphorylation, resulting in decreased proliferation.

## 4. Discussion

**Figure 9:**
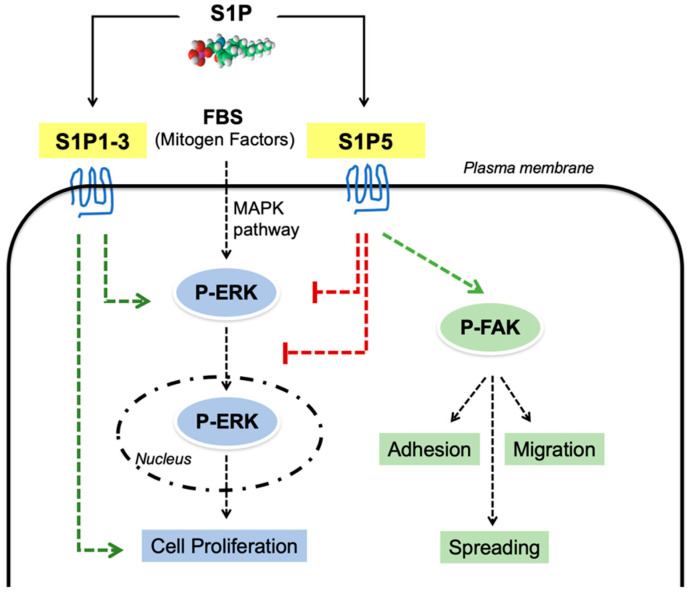
Model of S1P5 function in MEF cell proliferation, spreading and migration. Extracellular S1P (i) decreases FBS-induced ERK activation and subcellular localization via S1P5, (ii) promotes cell proliferation via S1P1-3, and (iii) induces FAK phosphorylation, resulting in cell adhesion, spreading, and migration.

The cellular functions of the S1P receptor S1P5 are still poorly characterized. It is well established that S1P5 affects the immune quiescence of the brain endothelial barrier, as well as natural killer cell trafficking [12,13,33]. Other studies have shown that in cancer cells, S1P5 regulates mitotic progression and autophagy [16,34], and S1P5 overexpression inhibits the growth of esophageal squamous cell carcinoma Eca 109 cells [15]. Thus, the current knowledge regarding the effects of S1P5 on cell proliferation and migration is limited and remains uncharacterized in untransformed cells. In this work, we investigated the effects of S1P5 deficiency in MEFs and showed that lack of S1P5 promotes immortalization and cell proliferation by controlling the spatial activation of ERK. We also found that, in S1P5^−/−^ MEFs, cell spreading, adhesion and migration are compromised and those phenotypes correlate with defective FAK activation (Figure 9).

S1P5 deficiency resulted in cell elongation and decreased cell surface area, while cell adhesion and spreading were compromised. The actin filament network and the focal adhesions (FA) are major regulators of cell spreading and morphology and initiate migration in various cell types, including fibroblasts [35]. MEF adhesion requires focal adhesion kinase (FAK), a highly conserved cytoplasmic enzyme, activated when integrins bind to ECM proteins, and in turn phosphorylated FAK activates several proteins including Rho GTPases involved in F-actin organization and dynamics [36,37]. Consistent with this model, S1P5^−/−^ cells exhibited fewer and sparser focal adhesion sites and increased cortical actin. A similar phenotype has been reported for FAK^−/−^ MEFs [38], supporting the idea that FAK acts downstream of S1P/S1P5 to control cell adhesion and spreading. It has been reported that S1P rapidly induces FAK phosphorylation in endothelial cells, but the involvement of one or more specific S1P receptors is not clear [39,40,41]. Here we show for the first time that S1P-mediated FAK phosphorylation is mediated by S1P5 and that, in S1P5^−/−^ cells, FAK activation is inhibited, which correlates with morphological changes and spreading defects. Finally, S1P5 positively regulated MEF migration. In agreement with our results, it was reported that Eca 109 esophageal cancer cells expressing GFP-S1P5 migrate faster than control cells under basal culture conditions [15]. Further studies are needed, including loss-of-function strategies, to decipher the role of S1P5 in cancer cell migration and the role of actin regulators such as Rho GTPases downstream of S1P5.

Converging evidence suggests that S1P5 negatively regulates cell proliferation, but the underlying molecular mechanism remains unknown. Overexpression of S1P5 in Eca 109 (esophageal cancer cells) and CHO cells results in growth inhibition [15,29]. Here we show that primary and immortalized S1P5^−/−^ MEFs proliferate faster and form more colonies than wild-type cells, suggesting a tumor suppressor function for S1P5. Of note, S1P1-3 receptors promoted MEF proliferation while S1P/S1P5 signaling inhibited cell growth, suggesting that activation of a specific subset of receptors by S1P can induce different signaling pathways leading to opposite cell responses. In CHO cells overexpressing S1P5, S1P inhibits serum-induced phosphorylation of ERK [29] and the authors proposed that the phosphatase PP2A acts downstream of the S1P/S1P5 pathway to limit ERK activation. ERK signaling plays a crucial role in various cellular functions such as cell proliferation, differentiation and survival. According to the current model, the spatial control of ERK activation (differences in magnitude as well as in subcellular compartmentalization) would explain distinct fates [32,42]. This spatial control of ERK activity is necessary for the control of MEF growth, with nuclear phospho-ERK leading to cell cycle progression, while the cytoplasmic phospho-ERK promotes cell cycle arrest [32,43]. Here we show that the absence of S1P5 results in sustained ERK activation when cells are grown in the presence of mitogenic factors and correlates with increased ERK activation in the nucleus. Activation of ERK by mitogenic factors is required for its nuclear translocation and nuclear activities [44], while its inactivation is mediated by the nuclear MAPK-specific phosphatases MKP1 and MKP2 [45]. An attractive hypothesis is that S1P5 may limit ERK nuclear localization by activating nuclear phosphatases, triggering ERK inactivation and subsequent cytoplasmic translocation. Alternatively, the S1P/S1P5 axis could cause the cytoplasmic retention of ERK by a yet unclear mechanism. In this regard, several spatial regulators such as β-arrestin, Sef or paxillin have been reported to sequester ERK activity in the cytoplasm [32].

S1P receptors are expressed in almost all cell types and can couple to multiple G proteins (G_i_, G_q_, and G_12/13_) to mediate a wide range of responses. Based on the current state of knowledge, S1P1 appears to selectively couple to G_i_ family members, S1P2 and S1P3 couple to G_i_, G_q_, and G_12/13_ members, whereas S1P5 couples to G_i_ and G_12/13_ but not to G_s_ or G_q_ [46]. Direct coupling of S1P5 to G_i_ was demonstrated by PTX-sensitive inhibition of adenylyl cyclase activity by S1P in Rh7777 hepatoma cells and rat receptor-overexpressing CHO cells [29,47]. However, while S1P1-3 induces G_i_-mediated activation of ERK to promote cell proliferation, in CHO cells expressing S1P5, S1P does not activate ERK [29]. In addition, S1P5 inhibits serum-induced activation of ERK1/2, but it remains to be determined whether this effect is mediated by specific G proteins downstream of S1P5. On the other hand, GPCR signaling via G_12/13_ activates Rho GTPases, well-known regulators of actin organization that affect cell shape, adhesion and migration [48]. The coupling of S1P5 to G_12_ has been demonstrated in CHO cells overexpressing the receptor [29], however functional data for coupling of S1P5 to G_12_ (and G_13_), such as the activation of Rho proteins, remains to be obtained. The loss of S1P5 in MEFs inhibits S1P-induced FAK activation, resulting in reduced cell spreading and migration. One hypothesis is that S1P-induced G_12/13_-RhoA signaling leads to FAK activation. Such a mechanism has been previously reported for lysophosphatidic acid (LPA)-stimulated cancer cell migration [49]. Interestingly, inhibition and stimulation of Rho proteins by S1P has been reported not only via G_12/13_ proteins but also through G_i_ proteins [50,51]. These results indicate that integration of opposing signals from the G_i_- and the G_12/13_-Rho pathways directs either positive or negative regulation of Rho proteins, and thus cell migration, upon activation of a single S1P receptor isoform.

It is well established that Sphingosine Kinase 1 is overexpressed or hyperactivated in a variety of tumors, leading to increased S1P production and secretion locally [52]. As a consequence, such an S1P-enriched tumor microenvironment affects growth and survival properties of tumor cells depending on the expression repertoire of S1P receptors. Future studies are needed to first evaluate the status of S1P5 expression and activity in cancer cell lines and tumor samples, and second to assess how loss of S1P5 function might be involved in tumor initiation and/or progression by promoting immortalization and proliferation.

This study indicates that S1P5 deficiency results in a bypass of replicative senescence in primary MEFs leading to spontaneous immortalization. Two canonical pathways, p53-p21 and pRB-p16, are functionally inactivated and allow cells to escape senescence-related growth arrest [53,54]. It will be interesting to investigate the molecular mechanism underlying the role of S1P5 in senescence and to evaluate the role of the S1P/S1P5 pathway in oncogene- or DNA damage-induced cellular senescence.

## 5. Conclusions

Our study reveals that S1P5 deficiency promotes immortalization and increases cell proliferation. Mechanistically, S1P5 negatively regulates the spatial activation of ERK limiting cell proliferation, and promotes the activation of FAK, thereby controlling cell spreading and adhesion.

## Data Availability

Data sharing not applicable.

## Supplementary Materials

The following supporting information can be downloaded at: https://www.mdpi.com/article/10.3390/cancers14071661/s1, Figure S1: Actin organization and FA in S1P5^−/−^ MEFs, Figure S2: FAK phosphorylation is decreased in S1P5^−/−^ MEFs.
