# Supplementary material for: Sphingosine 1-Phosphate Receptor 5 (S1P5) Deficiency Promotes Proliferation and Immortalization of Mouse Embryonic Fibroblasts
Source: Cancers (Basel). 2022 Mar 25;14(7):1661. doi: 10.3390/cancers14071661 (PMC8996878; doi:10.3390/cancers14071661)
Supplement: Supplementary file 1 [file cancers-14-01661-s001.zip › cancers-1639132-supplementary.pdf]

cancers

MDPI

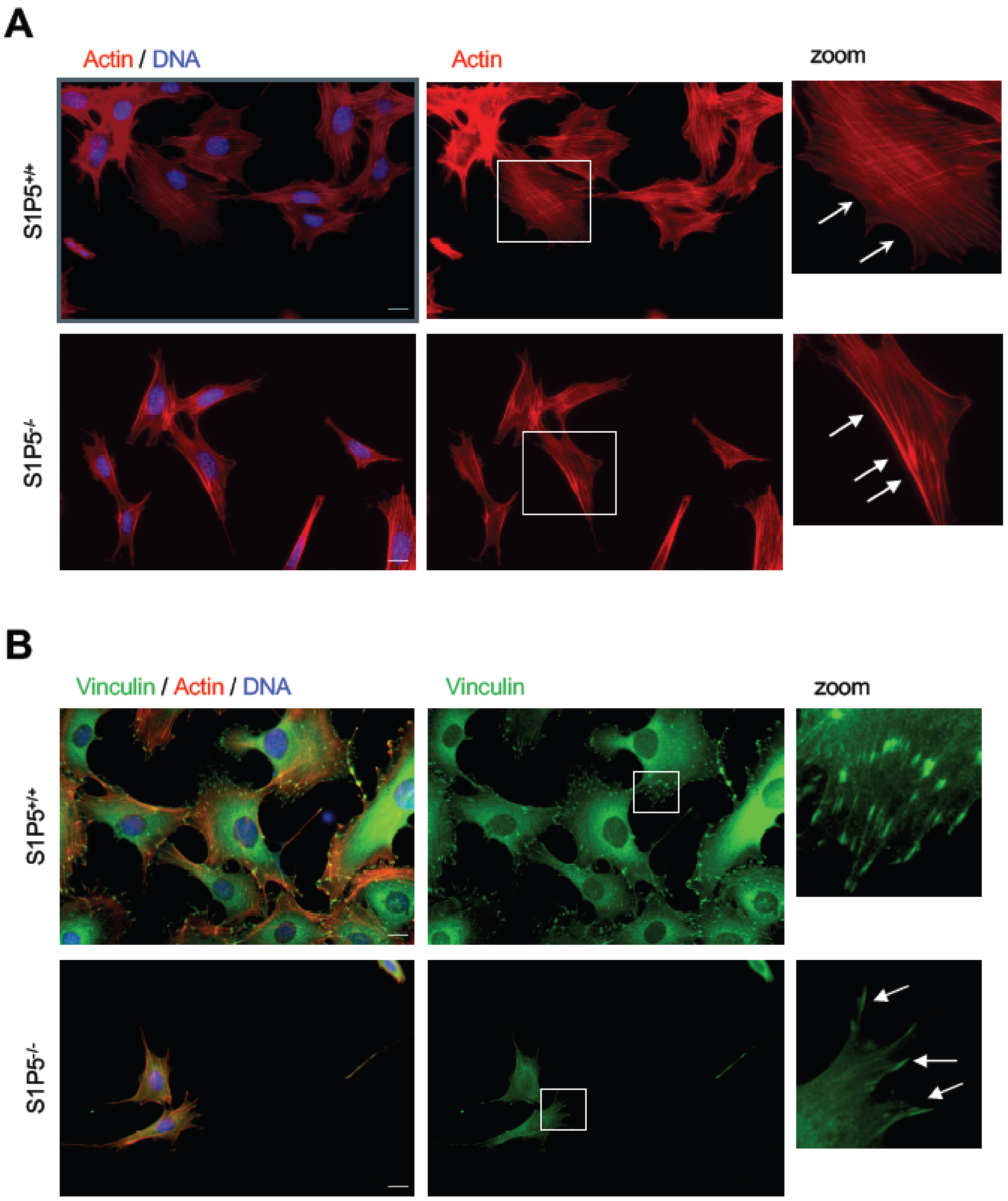

Figure S1. Actin organization and FA in S1P5-/- MEFs. (A), Representative images of S1P5+/+ and S1P5 -/- MEFs cultured in medium containing 10% FCS. Cells were fixed 24 h post plating and stained for F-Actin (Phalloidin-red) and DNA (Hoechst 33342-blue). Scale bar, 10 µm. (B), Representative images of S1P5+/+ and S1P5 -/- MEFs cultured as in A and stained for F-Actin (Phalloidin-red), vinculin (a-vin-green) and DNA (Hoechst 33342-blue). Scale bar, 10 µm.

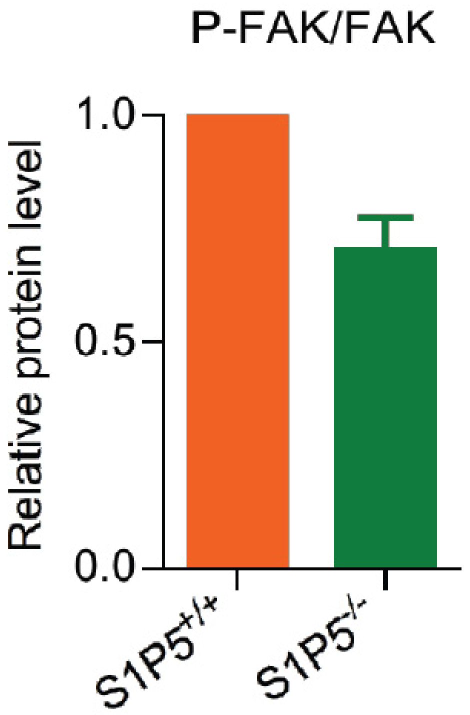

Figure S2. FAK phosphorylation is decreased in S1P5-/- MEFs. S1P5+/+ and S1P5 -/- MEFs were cultured in medium containing 10% FCS during 24 h, and the total cell extracts were analyzed by SDS/PAGE with antibodies against phosphorylated FAK, FAK and tubulin. Data represent the quantification of relative P-FAK/FAK levels of 3 independent experiments (means +/- SEM).
