# Supplementary figures and images for: Sphingosine 1-Phosphate Receptor 5 (S1P5) Deficiency Promotes Proliferation and Immortalization of Mouse Embryonic Fibroblasts
Source: Cancers (Basel). 2022 Mar 25;14(7):1661. doi: 10.3390/cancers14071661 (PMC8996878; doi:10.3390/cancers14071661)

# Original images for Western blots

Figure 3E

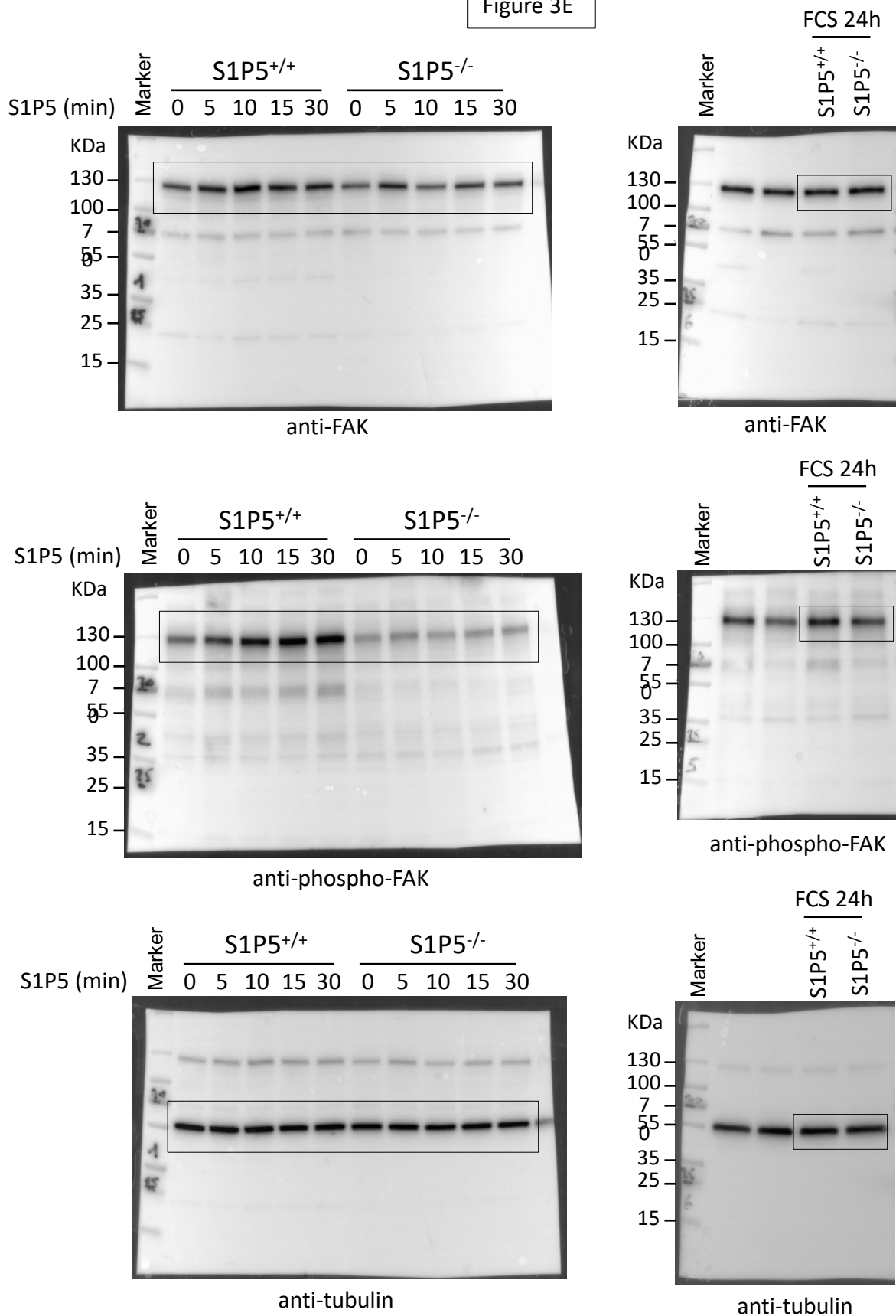

Figure 8A

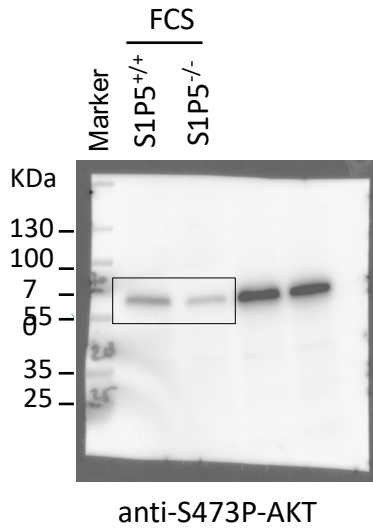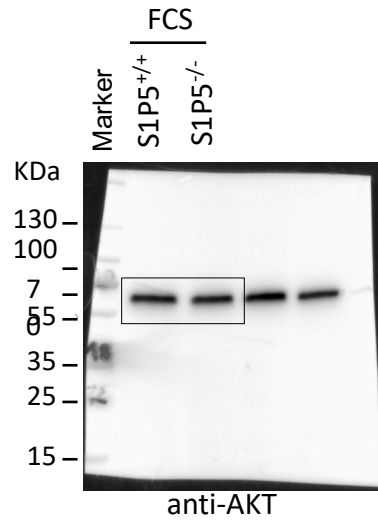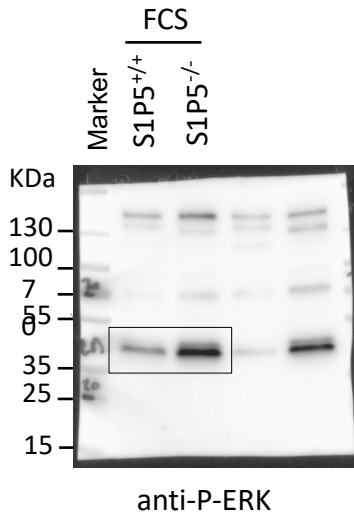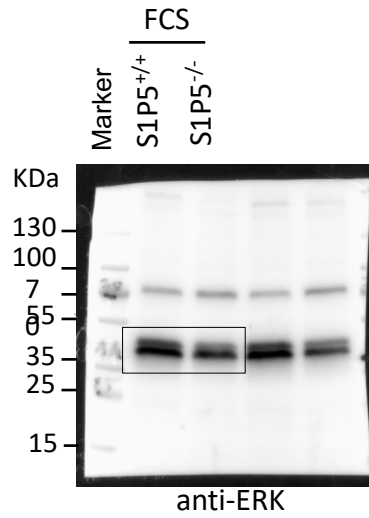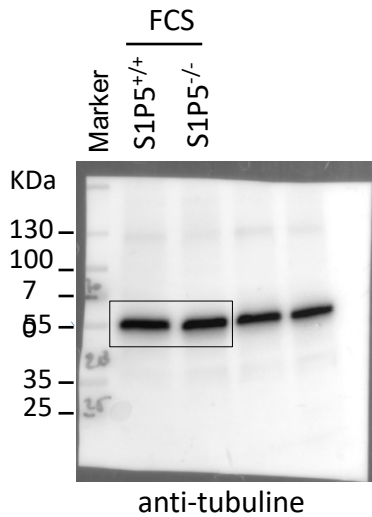

Supplement: Supplementary file 1 [file cancers-14-01661-s001.zip › cancers-1639132-original-images.pdf]
